# Global Distribution and Genetic Heterogeneity of Border Disease Virus

**DOI:** 10.3390/v13060950

**Published:** 2021-05-21

**Authors:** Cecilia Righi, Stefano Petrini, Ilaria Pierini, Monica Giammarioli, Gian Mario De Mia

**Affiliations:** Istituto Zooprofilattico Sperimentale dell’Umbria e delle Marche “Togo Rosati”, 06126 Perugia, Italy; c.righi@izsum.it (C.R.); s.petrini@izsum.it (S.P.); i.pierini@izsum.it (I.P.); m.giammarioli@izsum.it (M.G.)

**Keywords:** border disease virus, genetic heterogeneity, global distribution, genotypes, phylogenetic analysis

## Abstract

Border disease virus (BDV) belongs to the genus *Pestivirus* of the family *Flaviviridae*. Interspecies transmission of BDV between sheep, cattle, and pigs occurs regularly, sometimes making diagnosis a challenge. BDV can yield substantial economic losses, including prenatal and postnatal infections in lambs, which are the primary source of infection and maintenance of the virus in the population. Since BDV is antigenically and genetically related to bovine viral diarrhea virus (BVDV), it might pose a significant risk to cattle, influencing BVDV eradication campaigns. Similarly, the presence of BDV in swine herds due to pestivirus spillover between small ruminants and pigs might cause uncertainty in classical swine fever virus (CSFV) diagnostics. Therefore, knowledge of BDV epidemiology in different geographical regions will help prevent its spread and optimize control measures. Previous epidemiological studies have shown that various BDV genotypes are predominant in different countries. This review provides an overview of the spread of BDV world-wide in different host species.

## 1. Introduction

Border disease virus (BDV) is an important pathogen of sheep, and occasionally goats, reported globally, responsible for congenital disorders, abortion, stillbirths, birth of weak lambs experiencing tremors, abnormal body conformation, hairy fleece, immunosuppression, and increased risk of other infections, incurring substantial yet underestimated economic loss to the livestock industry [1]. The first case of BDV infection was reported in sheep in 1959 from the border regions of England and Wales [2]. Its seroprevalence may vary from 5% to more than 90% among adult sheep, depending on the management of animal husbandry, while mortality depends on the timing of infection, virulence of the infecting strain, and the species or breed of the infected host [3]. Clinical manifestations are usually mild in acutely infected sheep; nevertheless, they may range from asymptomatic to clinically severe. In 1984, a high mortality BDV strain, the Aveyron strain, was isolated from sheep from the Aveyron region of France [4,5]. In 1997, a horizontal BDV outbreak, characterized by high mortality, with clinical signs similar to those of the Aveyron disease, was observed in lambs in a flock in northeast Spain [6]. In 2001, in the Central and Eastern Pyrenees, a fatal BDV epidemic occurred in chamois (*Rupicapra pyrenaica pyrenaica*) causing a dramatic decrease (over 80%) in the chamois populations that threatened the species with extinction [7,8,9].

BDV belongs to the genus *Pestivirus,* family *Flaviviridae,* which comprises four major species, namely bovine viral diarrhea virus type 1 (BVDV-1), type 2 (BVDV-2), classical swine fever virus (CSFV), and border disease virus (BDV), and a growing number of additional putative *Pestivirus* species from various domestic and wild animals. Recently, a revision of the taxonomy of the genus *Pestivirus* was proposed, and the original members were reclassified into 11 viral species named from A through K [10,11]. A number of unclassified pestiviruses have been identified in various bovine species (*Pestivirus* H, atypical ruminant *pestivirus*, or HoBi-like viruses) and of non-bovine species such as antelope (*Pestivirus* E, Pronghorn antelope *pestivirus*), pigs (*Pestivirus* F, Bungowannah virus; *Pestivirus* K, atypical porcine *pestivirus* (APPV)), small ruminants (*Pestivirus* I, Aydin-like *pestivirus*), and giraffes (*Pestivirus* G or Giraffe 1).

Current segregation of BDV field isolates from sheep and goats, and the designation of genotypes by genetic analyses indicate that BDV can be phylogenetically identified into at least eight genotypes, from BDV-1 to BDV-8 [12,13,14,15,16]. Other ovine pestiviruses have been identified that are responsible for BD-like syndromes, indicating a separate evolutionary history that forms distinct genetic groups. In particular, the Tunisian, Tunisian-like, and Aydin-like (*Pestivirus* I, Turkey) pestiviruses are phylogenetically closer to CSFV than to BDV [17,18,19,20,21]. Recently, a new emerging ovine *pestivirus* (OVPV), highly divergent from known *pestivirus* species, was found to be genetically and antigenically closely related to CSFV [22,23], and could be defined as a novel species. The occurrence of BDV infection in domestic and wild animals, mostly in sheep, has been confirmed in different countries world-wide, but most of the data comes from Europe.

Several studies have reported the adaptive plasticity of BDV to cross the species barrier and infect pigs [24,25,26,27] and cattle [28,29,30,31,32,33,34,35,36,37,38]. Moreover, BDV can be transmitted among domesticated species and from domesticated to wild species such as chamois, llama, alpaca, bison, and reindeer [28,39,40,41]. Table 1 shows the segregation of BDV genotypes detected according to their host origin.

Usually, transmission is due to direct contact with infected animals via the oronasal route. Vertical transmission via the placenta plays an important role in the epidemiology of the disease [54]. Recently, BDV was isolated from *Melophagus ovinus*, a blood-sucking ectoparasite of sheep, collected from the body surface of sheep in Xinjiang (China), emphasizing the potential role of *Melophagus ovinus* as a carrier of BDV [55]. Moreover, some authors have identified the European hare (*Lepus europaeus*) to be a potential wild reservoir of *pestivirus* for livestock, particularly associated with high mortality outbreaks in Pyrenean chamois [56].

Clinical signs also include the birth of weak lambs and persistent infections (PI) in newborns because fetuses are exposed to the virus with an immature immune system [3,57]. These PI lambs, usually those with the “hairy shaker syndrome” (longer and finer coat), are viremic, antibody negative, and shed virus all their life, becoming the main source of infection in a flock [14,58,59]. To control the spread and prevent the disease, it is important to identify and separate such animals so that they will not be bred or traded, along with implementing biosecurity measures.

Based on their effects on susceptible tissue cultures similar to BVDV, two biotypes can also be distinguished in the BDV species: cytopathogenic (cp), which causes vacuolization and lysis of infected cells, and non-cytopathogenic (ncp), which does not lead to microscopically visible signs of infection [60,61]. The ncp biotype is isolated much more frequently than the cp biotype [51,62]. The emergence of cp strains in PI animals is crucial for the induction of fatal mucosal disease which, however, occurs rarely in sheep [3,63,64,65].

Currently, there are no proven effective vaccines for BDV, although in the USA and some European countries, killed whole-virus vaccines have been produced but are not commercially available [57,66]. Occasionally, attempts have been made to use BVDV vaccines, but they are ineffective because the two viruses are antigenically related but yet distinct and, therefore, do not confer full cross-protection [67].

## 2. Genomic Organization of BDV

BDV is an enveloped virus with a spherical shape (40–60 nm), and its genome consists of a single-stranded, positive polarity RNA, composed of approximately 12,300 nucleotides. BDV is easily inactivated by heat, drying, detergents, and UV light [68]. A single open reading frame (ORF) of the BDV genome encodes four structural proteins, the capsid (C) and three envelope glycoproteins (E^rns^, E1, and E2), and seven to eight non-structural proteins (N^pro^, p7, NS2–3, NS4A, NS4B, NS5A, and NS5B), which are flanked by 5′ and 3′ large untranslated regions (UTRs) [69,70,71]. Currently, only 15 complete BDV genome sequences are publicly available, representing each genotype, with the exception of BDV-6. The full genome sequence alignment displayed an intra-species similarity of 72.9% to 74.1% and 78.6% to 82.7% at nucleotide and amino acid sequence levels, respectively.

Pestiviruses were designated according to the host species, localization in specific tissues and organs, transmission route and symptoms of the disease, year of collection, and region of origin [72]. Nevertheless, these criteria were not satisfactory because there is extensive cross-reactivity and interspecies transmission among pestiviruses [12]. Subsequently, BDVs were identified by their genetic and antigenic relatedness to other viral strains and by the original host [10,14,39,57,73]. Previously, pestiviruses were phylogenetically characterized by comparing the 5′-UTR, N^pro^, and E2 gene sequences to classify new virus isolates. The 5′-UTR is the most conserved region of the viral genome, and the non-structural protein, N^pro^, codes for the N-terminal autoprotease that has no counterpart in other flaviviruses, whereas the E2 protein plays a major role in virus attachment and entry and is also important to induce neutralizing antibody production. To date, genotyping using 5′-UTR, N^pro^, and E2 sequences has provided consistent results to group isolates using phylogeny [12,15,45,74,75,76], and to define primers for identification and amplification of all pestiviruses by RT-PCR [77,78].

## 3. Global Distribution of BDV Genotypes

Several epidemiological studies have shown that BDV genotypes can affect a wide range of ungulates, but generally, sheep seem naturally sensitive to pestivirus infection. BDV infection is globally distributed and was reported in different European countries such as Austria [30,32,36,46,79,80], France [4,5,14,41,81], Germany [12,39,44,82,83], Italy [15,16,31,66,76,77,78,79], the Netherlands [24,28,84], Slovakia [85], Spain [1,6,7,9,13,27,48,86,87,88,89,90,91,92,93,94,95], Switzerland [35,51,92,96,97,98,99], and the United Kingdom [3,12,33,72,100].

BDV has also been reported outside of Europe, in Australia [28,70,92], China [101,102], India [103], Japan [25,26], Mexico [37], New Zealand [34,42,72], Tajikistan [104], and the USA [43,65,105]. In North America, in the 1970s, a disease characterized by unthriftiness, hairy fleece, and tremors in fetuses and newborn lambs was reported in northern California [106]. Until then, no sequence data from North American ovine isolates encompassing the entire structural region of a “true BDV” were published, and it was not clear whether true BDV-like viruses existed in this part of the world. Several years later, Sullivan et al. (1997) [43] retrospectively analyzed the entire structural gene coding region of an ovine pestivirus named BD31, originating from the flock in California, and found that this pestivirus was in fact a “true BDV”. This was the only reported detection of BDV in North America until 2012, when another case of BDV infection was reported in California [65]. Similarly, there were no reports on BDV circulation in South America, despite the fact that BD-like syndromes were observed in some places (personal communications). It is likely that BDV is present in other European and non-European countries where the size and density of small ruminant population are sufficient to allow disease circulation. On the other hand, the owners of small ruminant flocks are generally unaware of the disease, mostly because the clinical symptoms are not strongly obvious; therefore, they often go unrecognized.

High seroprevalence from 30% to 98%, depending on the geographical area, was reported. Indeed, a number of epidemiological reports concern only the seroprevalence of BDV infection in many countries, such as Austria [46], Chile [107], Denmark [108], Iran [109], Iraq [110], Ireland [111,112], Israel [3], the Netherlands [84], Peru [113,114], Sweden [115], Switzerland [40,96], the UK [116], and Mediterranean countries such as Algeria [117], Morocco [118], Tunisia [119], and Turkey [19,120]. Nevertheless, only a few cases were confirmed by virus isolation or molecular characterization. Fihri et al. (2019) [118] have reported that genome and antigen absence in RT-PCR of samples of animals from endemic areas could be explained by partial or total destruction of viral proteins during sample handling, shipment, and storage, or could also be due to early death of PI animals.

Serology is an ineffective tool because of the antigenic similarity of BDV with other related pestiviruses and the cross-infection of cattle, sheep, goats, pigs, and of some non-domesticated species. Indeed, BVDV and BDV are frequently detected in cattle, sheep, goats, and pigs, with low species specificity in serological tests. The transmission of pestiviruses between different animal species is often associated with common pasture or other forms of close contact between animals [1,13,14]. In such conditions, the virus can rapidly evolve by introducing mutations in the gene sequence that could lead to emergence and spread of new genetic variants.

Although it was virtually impossible to monitor temporal changes in the presence of genotypes in various countries, antigenic and genetic classification of isolates from different geographical regions is essential to improve knowledge on the epidemiology of BDV. According to the International Committee on Taxonomy of Viruses (ICTV), the pestivirus species are demarcated using a range of criteria, including complete coding nucleotide sequences and deduced amino acid sequence relatedness, antigenic relatedness, and host of origin [10,12,73]. While some independent studies have analyzed the same BDV isolates with consistent results of segregation into genotypes, in other cases, the lack of standardization and the use of different genomic regions have led to conflicting results for some BDV isolates.

**Figure 1:**
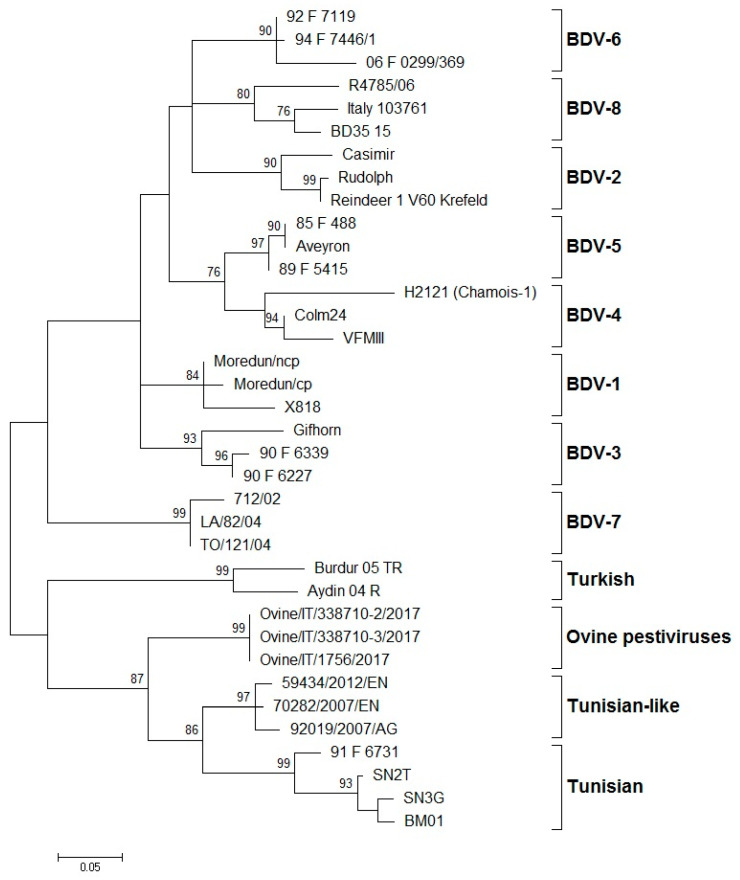
Phylogenetic tree showing the genetic relationship between pestivirus strains in the 5′-UTR. The tree was based on an analysis of partial 225 nt-long sequences. It was prepared using the MEGA v.7 program employing the maximum-likelihood method with the GTR+G+I substitution model. GenBank accession numbers of sequence data used are: BDV-1: X818 (AF037405), Moredun cp (U65022), Moredun ncp (U65023); BDV-2: Reindeer-1 V60 Krefeld (AF144618), Casimir (AB122085), Rudolph (AB122086); BDV-3: Gifhorn (EU636997), 90-F-6227 (EF693989), 90-F-6339 (EF693992); BDV-4: H2121 Chamois-1 (GU270877), VFMIII (DQ361071), Colm24 (DQ361073); BDV-5: Aveyron (KF918753), 89-F-5415 (EF693988), 85-F-488 (EF693985); BDV-6: 06-F-0299/369 (EF694001), 92-F-7119 (EF693994), 94-F-7446/1 (EF693996); BDV-7: 712/02 (AJ829444), LA/82/04 (FM163383), TO/121/04 (AM900848); BD-8: Italy-103761 (KT072634), BD35–15 (MF102262), R4785/06 (MF102260); Turkish: Aydin/04-TR (NC_018713), Burdur/05-TR (AM418428); Ovine pestiviruses: Ovine/IT/338710–2/17 (MK618725); Ovine/IT/338710–3/17 (MK618726); Ovine/IT/1756/17 (MG770617); Tunisian-like: 59434/2012/EN (KU856555), 70282/2007/EN (KU856551), 92019/2007/AG (KU856552); and Tunisian: BM01 (AY453630), SN2T (AF461996), SN3G (AY583306), 91-F-6731 (EF988632). Numbers indicate the percentage of 10,000 bootstrap replicates that support each phylogenetic branch. Bar: number of substitutions per site.

The systematic typing of the BDV isolates, based on the 5′-UTR, Npro, and E2 regions, led to the identification of eight phylogenetic groups (BDV-1 to BDV-8) (Figure 1).

The country-wise segregation of BDV isolates into genotypes is summarized in Table 2.

BDV-1, with putative subgroups BDV-1a and BDV-1b, and BDV-2, which consists solely of German isolates, were originally referred to as BDV-A and BDV-B, respectively [72]. BDV-3 is the most prevalent genotype in Europe, followed by BDV-1, which is prevalent world-wide; BDV-4, with probable subgroups BDV-4a and BDV-4b [1], originally referred to as BDV-C by Hurtado et al. (2003) [86], is predominant in Spain. BDV-5 and BDV-6 were first reported in France [14].

In 2005, the first member of a putative novel *pestivirus* group, an atypical *pestivirus*, was detected in Italy [49]. Further comparative analysis of small ruminant *pestivirus* sequences confirmed the evidence of the novel genotype BDV-7 [15,50], which is currently only detected in Italy. Interestingly, the near-complete nucleotide sequence so far available within this BDV-7 genotype (LR824489) showed an intra-species nucleotide (nt) and amino acid (aa) *p-distance* slightly exceeding the values 0.24 and 0.15, proposed by Smith et al. (2017) [10] to be the genetic criterion for the demarcation of the BDV species, thereby placing the BDV-7 group slightly farther outside than the other BDV genotypes [121].

BDV-8 was subsequently reported by Peletto et al. (2016) and Caruso et al. (2017) [16,52], respectively, from a goat kid and a chamois in Italy, and by Peterhans et al. (2010) [51] and Stalder et al. (2017) [53] from cattle, sheep, and pigs in Switzerland. In the N^pro^ genomic region, the sequences of the strains reported from Italy matched 82–92% (nt) and 86–93% (aa) to the corresponding region in the Swiss strains.

**Figure 2:**
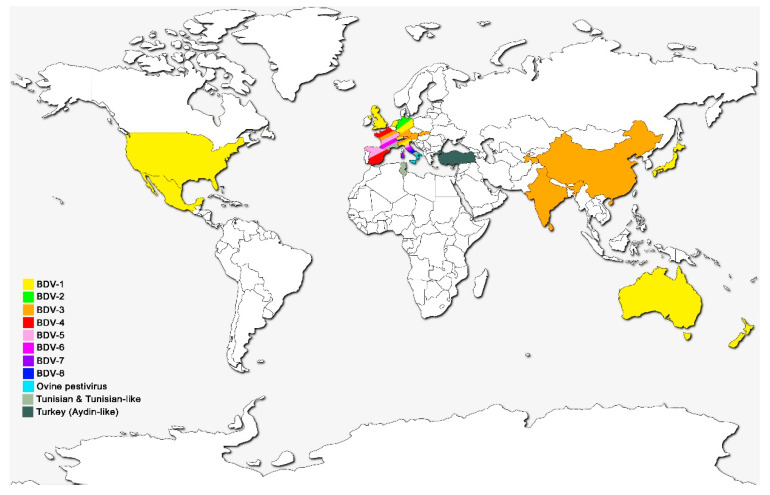
Global geographical distribution of BDV genotypes.

**Figure 3:**
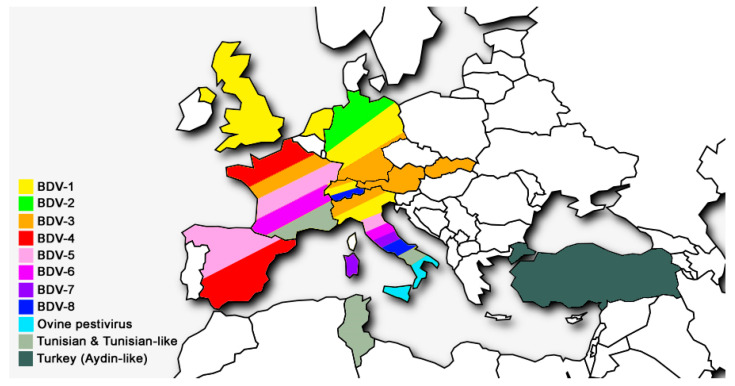
Geographical distribution of BDV genotypes in Europe and in the Mediterranean basin.

It is also noteworthy that Italy is the country where the highest number of BDV genotypes has been documented, indicating a greater genetic diversity of the virus compared to other countries. However, the data are insufficient to draw conclusions about the overall epidemiological situation of BDV infection, since data from countries in Central and Eastern Europe, Africa, and South America are still missing (Figure 2 and Figure 3).

## 4. Conclusions

In previous years, border disease often went unnoticed, as its symptoms, mainly related to fertility, rather than morbidity or mortality in animals, were not directly ascribed to BDV infection, and were sometimes confused with other pathogens, such as BVDV, *Chlamydophila* spp., *Campylobacter* spp., *Salmonella* spp., *E. coli*, and *Corynebacterium* spp. It affects reproduction, reduces growth rate, and hence delays slaughter in commercial conditions, yielding economic loss. Molecular investigations represent a useful tool to prevent and control the spread of disease, and to identify the changes in genetic characteristics of isolates by monitoring *pestivirus* strains in ruminants, particularly in mixed herds. Molecular epidemiology is instrumental in classifying novel viruses and studying their evolutionary dynamics. Phylogenetic analysis of BDV seeks to establish epidemiological patterns by tracing globally circulating virus isolates to individual countries. This improves our understanding of pathogenesis and infection routes, establishes genetic relationships between different isolates, and evaluates their temporospatial distribution in animal populations. Additionally, due to the spread and risks of BDV, and the challenges in controlling the disease, determining the antigenic relationship among *pestivirus* isolates plays a major role in diagnosis, planning immunization strategies, and controlling infection.

This review aimed to provide comprehensive knowledge on the variability of BDV and the genetic relatedness among circulating strains, thus avoiding inconsistent or conflicting allocation of BDV isolates into genotypes. At least eight different BDV genotypes, spread world-wide, have been described so far. It can be speculated that this genetic variability and the similarity observed among some viruses isolated from distant countries might be associated, at least partially, with animal trade.

While genetic comparisons are useful, the relationship between genetic and antigenic differences is largely unknown. This prompts the question on whether the genetic diversity of field strains can affect the diagnostics. Previous studies [12] have demonstrated even significant antigenic variations between BDV-1, BDV-2, and BDV-3 strains. Nevertheless, the criteria by which membership of one genotype is assigned rather than another do not necessarily correlate with the antigenic characteristics.

Locally, a flock management program should aim to identify and remove all PI animals from the flock, and prevent contact with or introduction of BDV-infected animals from outside. It would be imperative to only purchase from BDV-free flocks or isolate all newly purchased animals until they can be tested free of BDV.

The circulation of BDV may also substantially influence BVDV and CSFV eradication measures [25]. There is evidence that BDV PI sheep could transmit infection to cattle during close contact, for example, while sharing communal pastures. Additionally, since many tests used for the detection of BVDV antibodies do not differentiate between BVDV and BDV infection, this could pose risks and uncertainty to BVDV control programs. Therefore, small ruminants, particularly pregnant animals, should not be ignored in eradication programs for cattle, and it would be necessary to develop more specific diagnostic tests to detect all the ruminant pestiviruses and discriminate among them to detect BDV in both cattle and sheep. This is also applicable to swine herds, where BDV might be confused with CSFV in CSFV eradication programs, which may result in the unnecessary slaughter of large numbers of CSFV-free animals.

Genetic changes in the BDV genome can alter virulence, and novel ovine pestiviruses can emerge that could impact the epidemiology of the disease. Therefore, it is essential to study the genetic diversity and unequivocally designate BDV genotypes, considering that the heterogeneity of pestiviruses will probably increase in the future. In many geographical regions, BDV circulation is not yet studied, which is necessary to obtain a comprehensive epidemiological picture of BDV infections, especially in countries where sheep and goat rearing is traditionally an important source of income.

## Figures and Tables

**Table 1:** BDV genotypes and other ruminant pestiviruses according to the host species.

Genotypes of BDV	Host Origin of Isolates	Country of Isolation	References
BDV-1	Alpaca, llama, sheep, cattle, goat, pig	Australia, Germany, Italy, Japan, Mexico, Netherlands, New Zealand, Switzerland, UK, USA	[28,40,42,43]
BDV-2	Reindeer, sheep, wisent	Germany	[12,44]
BDV-3	Sheep, goat, cattle	Austria, China, France, Germany, India, Italy, Slovakia, Switzerland	[14,31,45,46,47]
BDV-4	Chamois, sheep, pig	France, Spain	[7,13,27,48]
BDV-5	Sheep, goat	France, Italy, Spain	[6,14,45]
BDV-6	Sheep, chamois	France, Italy	[14,41]
BDV-7	Sheep, goat	Italy	[15,49,50]
BDV-8	Goat, chamois, sheep, cattle, pig	Italy, Switzerland	[16,51,52,53]
Turkey (Aydin-like)	Sheep, goat	Turkey	[19]
Tunisian and Tunisian-like	Sheep, goat	France, Italy, Tunisia	[17,18]
Ovine *pestivirus*	Sheep	Italy	[22]

**Table 2:** Global distribution of BDV genotypes and other small ruminant pestiviruses.

Country of Isolation	Genomic Region	Year of Isolation	Pestivirus D								Pestivirus I	Unassigned Pestiviruses		References
			BDV-1	BDV-2	BDV-3	BDV-4	BDV-5	BDV-6	BDV-7	BDV-8	Turkey (Aydin-Like)	Tunisian and Tunisian-Like	Ovine Pestivirus	
Australia	E1, E2	1973	2	-	-	-	-	-	-	-	-	-	-	[28,70,92]
Austria	5′-UTR, N^pro^	2003–2014	-	-	29	-	-	-	-	-	-	-	-	[30,32,36,46,79,80]
China	5′-UTR, Npro	2012	-	-	4	-	-	-	-	-	-	-	-	[101,102]
France	5′-UTR, N^pro^	1984–2010	-	-	7	2	5	11	-	-	-	3	-	[4,5,14,41,81]
Germany	N^pro^, E1, E2	1985–2001	1	6	1	-	-	-	-	-	-	-	-	[12,39,44,82,83]
India	5′-UTR, Npro, E2	2009–2010	-	-	1	-	-	-	-	-	-	-	-	[103]
Italy	5′-UTR, N^pro^	2002–2018	3	-	7	-	1	1(*)	14	2	-	3	4	[15,16,18,22,23,31,45,47,49,50,52]
Japan	5′-UTR, Npro, E2	2012	4	-	-	-	-	-	-	-	-	-	-	[25,26]
Mexico	5′UTR	2016	3	-	-	-	-	-	-	-	-	-	-	[37]
Netherlands	5′-UTR	1994	1	-	-	-	-	-	-	-	-	-	-	[24,28]
New Zealand	5′-UTR, Npro	1990–2010	5	-	-	-	-	-	-	-	-	-	-	[34,42,72]
Slovakia	5′-UTR, N^pro^, E2	2007	-	-	1	-	-	-	-	-	-	-	-	[85]
Spain	5′-UTR, N^pro^, E2	1996–2011	-	-	-	83	5	-	-	-	-	-	1	[1,6,7,13,27,48,86,87,89,90,91,92,93]
Switzerland	5′-UTR, N^pro^	2001–2006	1	-	1	-	-	-	-	5	-	-	-	[35,51,53,92,97,99]
Tajikistan	5′-UTR, N^pro^, E2	2017	-	-	1	-	-	-	-	-	-	-	-	[104]
Tunisia	5′-UTR, N^pro^	1995–2000	-	-	-	-	-	-	-	-	-	10	-	[17,92]
Turkey	5′-UTR, N^pro^	2004	-	-	-	-	-	-	-	-	2	-	-	[19,20,21]
United Kingdom	5′-UTR, N^pro^, E2, E1	1976–2008	28	-	-	-	-	-	-	-	-	-	-	[12,33,72]
USA	5′-UTR, E^rns^, E1	1978–2012	2	-	-	-	-	-	-	-	-	-	-	[43,65,105]
Total Number			50	6	52	85	11	12	14	7	2	16	5	

(*) Unpublished data, GenBank accession no. HG995106.

## Data Availability

Data that support the findings of this study are openly available in NCBI at www.ncbi.nlm.nih.gov.
